# Stage Specificity, the Dynamic Regulators and the Unique Orchid *Arundina graminifolia*

**DOI:** 10.3390/ijms222010935

**Published:** 2021-10-10

**Authors:** Sagheer Ahmad, Chuqiao Lu, Yonglu Wei, Jie Gao, Jianpeng Jin, Chuanyuan Zheng, Genfa Zhu, Fengxi Yang

**Affiliations:** Guangdong Key Laboratory of Ornamental Plant Germplasm Innovation and Utilization, Environmental Horticulture Research Institute, Guangdong Academy of Agricultural Sciences, Guangzhou 510640, China; sagheerhortii@gmail.com (S.A.); luchuqiao@gdaas.cn (C.L.); weiyonglu@gdaas.cn (Y.W.); gaojie@gdaas.cn (J.G.); jinjianpeng@gdaas.cn (J.J.); zcytrain@zhku.edu.cn (C.Z.); zhugenfa@gdaas.cn (G.Z.)

**Keywords:** bamboo orchid, stage specificity, flowering, hormonal regulation, transcriptome

## Abstract

Orchids take years to reach flowering, but the unique bamboo orchid (*Arundina graminifolia*) achieves reproductive maturity in six months and then keeps on year round flowering. Therefore, studying different aspects of its growth, development and flowering is key to boost breeding programs for orchids. This study uses transcriptome tools to discuss genetic regulation in five stages of flower development and four tissue types. Stage specificity was focused to distinguish genes specifically expressed in different stages of flower development and tissue types. The top 10 highly expressed genes suggested unique regulatory patterns for each stage or tissue. The *A. graminifolia* sequences were blasted in *Arabidopsis* genome to validate stage specific genes and to predict important hormonal and cell regulators. Moreover, weighted gene co-expression network analysis (WGCNA) modules were ascertained to suggest highly influential hubs for early and late stages of flower development, leaf and root. Hormonal regulators were abundant in all data sets, such as auxin (LAX2, GH3.1 and SAUR41), cytokinin (LOG1), gibberellin (GASA3 and YAB4), abscisic acid (DPBF3) and sucrose (SWEET4 and SWEET13). Findings of this study, thus, give a fine sketch of genetic variability in Orchidaceae and broaden our understanding of orchid flower development and the involvement of multiple pathways.

## 1. Introduction

Orchidaceae, one of the largest family of angiosperms, contains ornamental orchids [1,2]. More than 0.1 million orchid species are cultivated worldwide due to their immense horticultural importance. The most popular orchid species, such as Cymbidium and Phalaenopsis, flower in specific times of the year [3]. However, the bamboo orchid blooms year round and produces peak flowering from September to January. It is mainly found in sub-tropical and tropical areas of Asia [4,5,6,7]. It can grow on a variety of land and environmental conditions up to 2800 m altitudes, which shows its strong adaptability towards changing environment [7]. As a medicinal plant, it is a rich source of phenols, stilbenoids, bibenzyls and flavonoids, possessing antioxidant, anti-tumor and anti-viral properties [8]. The chemical constituents and medicinal compounds have been discussed in numerous studies, although the molecular pattering of floral regulation remains elusive for *A. graminifolia*.

Since antiquity, the plant hormones have been studied as important regulators of flowering in orchids [9]. Auxin is a morphogen and signalizes tissue specification through its concentration gradients [10,11,12,13,14]. Application of BA (6-benzylaminopurine) promotes flowering in orchids, such as Dendrobium and Phalaenopsis, but auxin counteracts this influence. However, BA is more effective in flowering regulation when applied with GA_3_ (gibberellic acid) [15]. Gibberellins play important roles in the regulation of stem elongation [16,17,18] and flowering time [19,20]. Abscisic acid (ABA) emerges as an important hormone regulating flowering time and bud break [21,22]. However, the exact role of ABA in flowering is not clear, as it exerts both positive and negative effects [21,22,23,24]. Environmental changes may be the driving force behind these contradictory effects [24,25]. Strigolactones are another class of hormones and their cross-talk with GA, ethylene, cytokinin and auxin influences plant growth and development through various pathways [26,27,28]. Despite the extensive involvement, the genetic underpinnings of hormonal effects on orchid flowering remain poorly understood.

Considering the importance of *A. graminifolia* for orchid breeding programs, we report the first de novo assembly for this orchid and discuss the stage specificity of differentially expressed genes for five stages of flower development and four tissue types. We elucidated highly expressed gene sets and hormonal and floral regulators in different stages of flower development, mature flower, capsule, leaf and root. WGCNA modules were generated for early and late FD stages, leaf and root. Moreover, a selective set of genes was experimentally validated through qRT-PCR. Therefore, this study enhances the understanding of orchid floral control at genetic levels and provides support to strengthen the breeding programs for orchids.

## 2. Results

### 2.1. A General Sketch of A. graminifolia

The juvenile phase of bamboo orchid completes in six months, which is quite fast as compared to other orchids, such as *Cymbidium* and *Phalaenopsis*, taking 2–3 years for vegetative growth. It flowers years round with vigorous flowering period between September and January. The whole reproductive period is 32.3 days and the ornamental duration of inflorescence is 188.2 days. On an average, a single plant bears 6.1 flowers (Supplementary Table S1).

Flower develops in six states, including Stage 0 to Stage 5. The development starts with flattened primordia (Stage 0), followed by appearance of floral buttress and undifferentiated division of primordia into different organ primordia (Stage 1). In the second stage a typical floral zygomorphy is established and floral organs differentiate. Then, sepals overlap the petals, showing an inverted triangle of floral apex (Stage 3). Rapid elongation of the gynostemium with immature pollinia represents Stage 4. In the last stage (Stage 5), the flowers open with a central column and four pollinia arranged around it (Supplementary Figure S1).

### 2.2. Transcriptome Sequencing and Functional Annotation

Transcriptomic analysis was performed to generate system level clues for different stages and tissues. Samples from five stages of flower development and four tissues (flower, capsule, leaf and root) produced 71.2 billion reads, with an average of 7.8 billion reads (10.8–12.8 Gb data) from each sample. We filtered 25,353 unigenes (with an average length of 1198 bp). These were annotated using GO, KEGG, Pfam, SwissProt, and eggNOG and NR databases (Supplementary Table S2). GO annotations were enriched in 17,171 unigenes and 9234 unigenes were annotated to KEGG categories. The highest number of unigenes (18,854) was annotated to eggNOG categories, followed by NR categories covering 72.29% of annotated unigenes.

### 2.3. Comparative Transcriptome among All Plant Parts Points Important Stage Determinants

**Figure 1:**
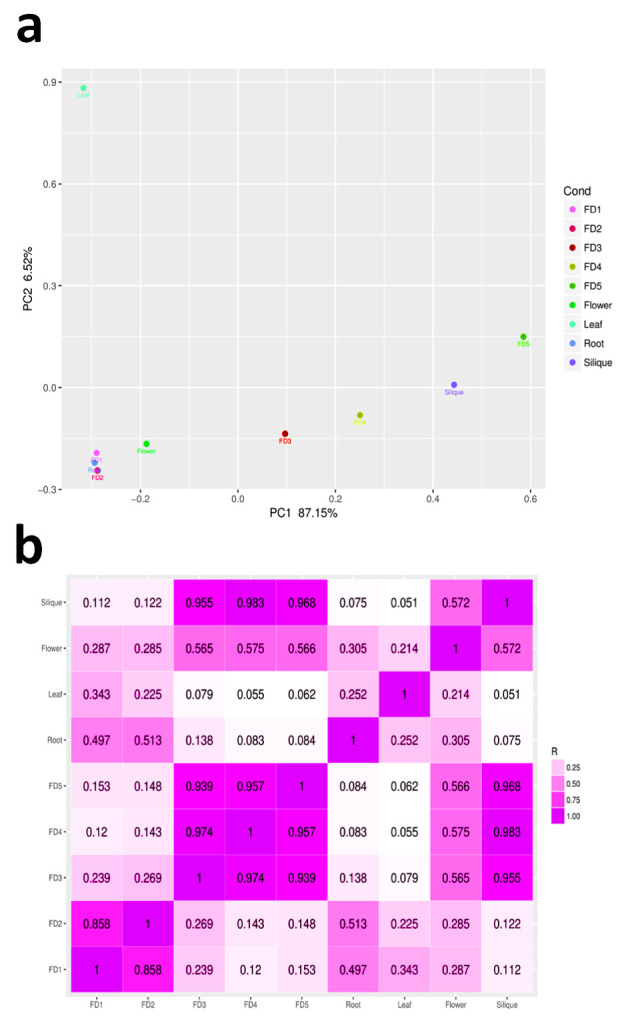
Principal component analysis (**a**) and Pearson correlation coefficient analysis (**b**).

In order to ascertain the transcriptomic differences in bud initiation and outgrowth dynamics among different plant parts, we carried out principal component analysis (PCA) and hierarchical clustering on the basis of spearman correlation coefficient (SCC) analysis of FPKM value of the genes expressing in at least one of the 3 tissues for each sample (Figure 1). It showed that first two stages of flower development clustered together, other three developmental stages were farther from this grouping. However, all of the floral developmental stages, flower and silique were clustered in the same pattern except leaf that occupied an opposite axis, showing marked difference from other plant parts (Figure 1a). Pearson correlation coefficient also points out important relationships between flower and other parts, and indicated that 4th stage of flower development shows the maximum affinity to flower (Figure 1b), suggesting that this stage might possess some pivotal regulators to trigger onset of flowering (R = 0.575).

### 2.4. Comparative Transcriptome among All Plant Parts Highlights Important Stage Determinants

**Figure 2:**
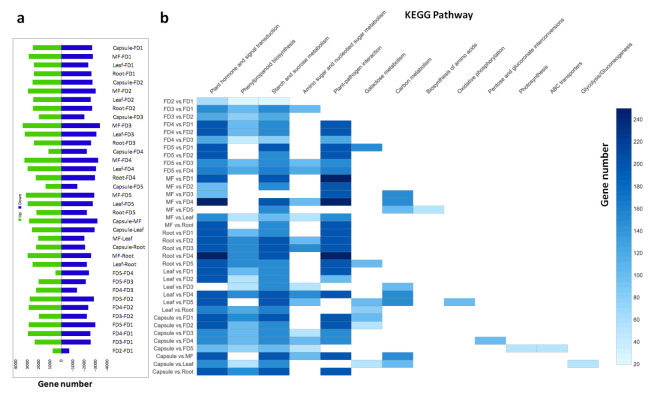
Comparison of up- and down-regulated DEGs among 36 pairs (**a**) and the abundance of highly enriched KEGG pathways (**b**).

We compared the samples in 36 pairs and identified DEGs between various combinations (Figure 2a). The comparison between mature flower and developmental stages 3–4 yielded the highest number of DEGs: FD3 and mature flower differed by 3383 upregulated and 3400 downregulated genes, and FD4 and mature flower differed by 3224 upregulated and 3254 downregulated genes. The highest number of DEGs (>200) were involved in plant hormone signal transduction, especially between mature flower and root, mature flower and capsule, and capsule and root. This was followed by DEGs involved in plant–pathogen interaction, and those acting in starch and sucrose metabolism (Figure 2b).

### 2.5. Stage-Specific Gene Sets during Bud Outgrowth and Positions

**Figure 3:**
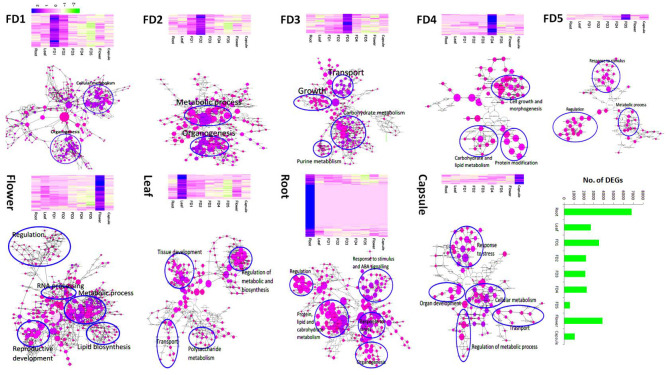
Heatmaps and cluster analysis of differentially expressed genes in each developmental stage and tissue type, and the number of stage specific DEGs.

The stage specificity (SS) algorithm identified DEGs with the highest expression for a particular developmental stage or tissue type. The highest numbers of DEGs (6846) were root specific (Figure 3). The fewest were associated with the 5th stage of flower development (FD5).

The gene ontology enrichment also showed variation in clustering patterns and specifications to biological processes for different plant tissues and developmental stages. FD1 and FD2 contained terms related to metabolic processing and organogenesis. FD3 and FD4 comprised terms related to growth, development, and carbohydrate, lipid, and protein metabolism. FD5 included terms relating to regulation and response to stimuli; and, mature flowers contained GO terms related to RNA processing, reproductive development, and metabolic processing. In leaf samples, tissue development-related genes clustered with transport and polysaccharide metabolism. The largest number of genes was clustered in root, specifying stress responses, and ABA signaling, organogenesis, and macro-molecule synthesis.

**Figure 4:**
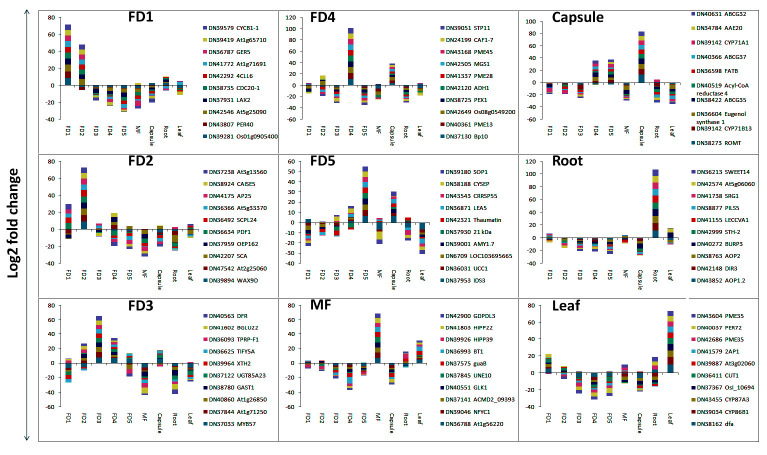
Top 10 highly expressed DEGsin each stage of flower development and tissue type.

Top 10 highly expressed DEGs specific to FDs and tissue types were sketched to elaborate their trends for tissue specificity (Figure 4). Auxin related DEGs (GER5, LAX2), homeobox protein (LET6) and cell division related proteins (CYCB1-1, CDC20-1) were evident in FD1. CAISE5 and At3g13560 were related to glucose metabolism in FD2. The genes upregulated in FD1 were also upregulated in FD2 in lower intensities and vice versa. MYB45 is a TF involved in GA-mediated signaling pathway (GO: 0009740), JA-mediated signaling pathway (GO: 0009867) and stamen filament development (GO: 0080086). It was specifically expressed in FD3. Moreover, an increasing and then decreasing trend can be seen in FD3, wherein gene expression grows from FD1, peaks in FD3 and then completely downregulates in mature flower. A distinct pattern of gene expression can be seen in root. The genes upregulated in root were completely downregulated in other tissues (Figure 4).

**Figure 5:**
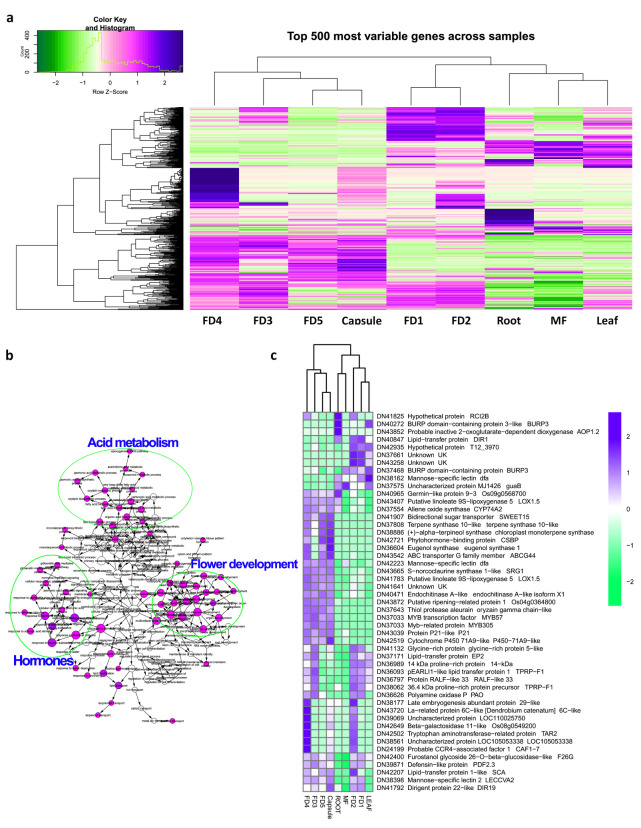
(**a**): Heatmap of the top 500 differentially expressed genes; (**b**):clustering of the top 50 DEGs by Cytoscape; (**c**):heatmap with gene names of the top 45 DEGs.

We used several R-packages (edgeR, limma, Glimma, gplots, org.Mm.eg.db and RColorBrewer) to filter the top 500 stage-specific genes with the highest expression values. FD1 and FD2 were clustered together and their expression patterns were much different as compared to other stages or tissue types (Figure 5a). We queried the protein sequences of top 50 DEGs genes against the *Arabidopsis* genome using Blast-2.10.0+ (https://ftp.ncbi.nlm.nih.gov/blast/executables/blast+/LATEST/) for blastp, and obtained the *Arabidopsis* locus IDs. We analyzed these IDs on Cytoscape (v3.8.2, National Resource for Network Biology, USA) (BINGO plug-in) to cluster genes with similar functions (Figure 5b). We identified gene clusters related to flower development, hormonal regulation, abscisic acid and gibberellic acid signaling, and important regulators of floral identity, such as MYB305, MYB57, TAR2, and ABA regulators (BURP3; Figure 5c).

### 2.6. Selective but Comprehensive Show of Stage and Tissue Determinants

**Figure 6:**
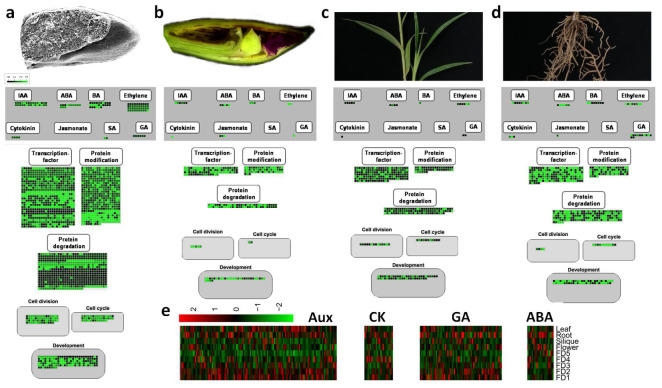
Morphological depiction, and hormonal regulation and cell cycle activities depiction of FD1 (**a**), FD4 (**b**), leaf (**c**) and root (**d**) using MapMan; (**e**) transcriptome data of major plant hormones.

Among the reproductive parts, FD1 was selected to analyze the start of flower development (Figure 6a) and FD4 was selected as a late state of flower development (Figure 6b). Leaf and root were selected as vegetative parts to compare with reproductive stages (Figure 6c,d). Upregulated genes to these particular stages and tissues were blasted against *Arabidopsis* genome using Blast-2.10.0+ platform. The corresponding *Arabidopsis* IDs with log2 fold change expressions were run on MapMan to see the regulation overview, and cellular response overview using the best *Arabidopsis* homolog (TAIR10). Regulation overview shows hormonal regulation, transcriptional activities, protein modification, and protein degradation. The cellular response overview includes cell division, cell cycle and development. High enrichment of important plant hormones can be seen in the first stage of flower development as compared to the late stage (Figure 6a,b). Intense transcriptional factor activities, protein modification and degradation are evident in the start of flower development. Similarly, elevated cell-level activities play role in the commencement of flower development. However, limited hormonal and cellular regulation can be seen in late stages of flower development. In the case of leaf and root, more hormonal and cellular activities are shown by root as compared to leaf (Figure 6c,d).

### 2.7. The Regulatory Hubs for Reproductive and Vegetative Tissues

**Figure 7:**
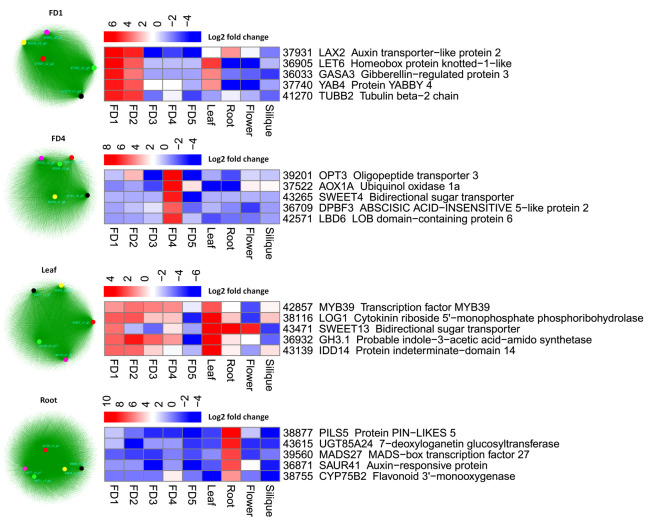
WGCNA-based clustering and identification of highly significant hub genes from each cluster. The heatmaps show the transcriptomic expressions of selected hub genes.

WGCNA modules help to elucidate highly correlated gene sets. We obtained co-expressed gene groups for an early stage of flower development (FD1), a late stage of flower development (FD4), leaf and root. The five highly correlated genes were selected from each stage-specific module as important hubs (Figure 7). Hormonal and sugar transport and signaling can be seen among the floral development stages. Auxin (LAX2) and gibberellin (GASA3) regulation genes were highly expressed in FD1 while abscisic acid (DPBF3) and sugar (SWEET4) signaling genes were more expressed in FD4. In the case of leaf, auxin (GH3.1), cytokinin (LOG1) and sugar (SWEET13) signaling can be seen along with transcription factors. The root showed regulation of auxin (SAUR41) responsive genes and flavonoid (CYP75B2 and UGT85A24) pathway genes (Figure 7).

### 2.8. Validation of Selected Genes by qRT-PCR

**Figure 8:**
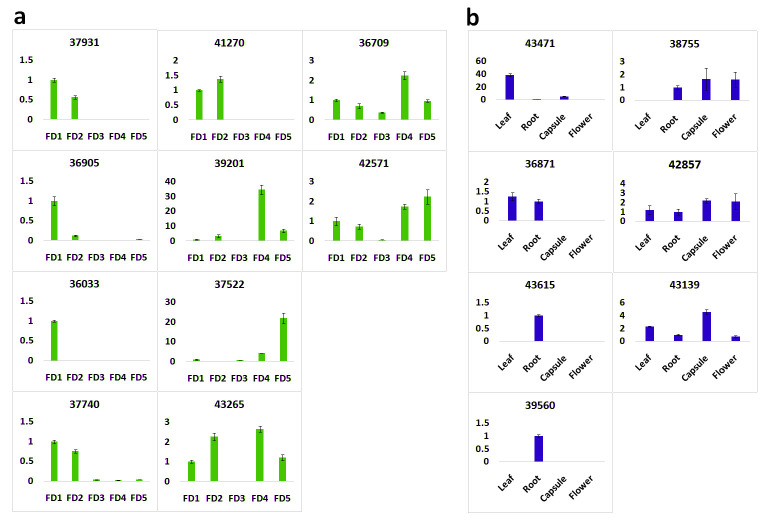
qRT-PCR expressions of selected genes in flower development (FD) stages (**a**) and vegetative tissues (**b**). FD1 to FD5 denotes the flower development stages 1–5. The genes expressed in flower development stages include, 37931 (auxin transporter-like protein), 36905 (Homeobox protein knotted-1-like), 36033 (gibberellin-regulated protein), 37740 (protein YABBY 4-like), 41270 (tubulin beta-2 chain), 39201 (oligopeptide transporter 3-like), 37522 (ubiquinol oxidase), 43265 (bidirectional sugar transporter), 36709 (ABSCISIC ACID-INSENSITIVE 5-like protein) and 42571 (LOB domain-containing protein). The genes expressed in vegetative tissues include, 43471 (bidirectional sugar transporter), 36871 (auxin-responsive protein), 43615 (7-deoxyloganetin glucosyltransferase), 39560 (MADS-box transcription factor 27), 38755 (Flavonoid 3’-monooxygenase), 42857 (Transcription factor MYB39) and 43139 (Protein indeterminate-domain 14).

Selective set of genes was validated for their tissue specificity through qRT-PCR. In FD1, the highest expression was observed for 37931 (auxin transporter-like protein), 36905 (Homeobox protein knotted-1-like), 36033 (gibberellin-regulated protein), and 37740 (protein YABBY 4-like) (Figure 8a). The 41270 (tubulin beta-2 chain) was fairly high in FD2 rather than FD1, but it cannot make much difference as FD1 and FD2 clustered together and there is no big difference of gene expressions between these two stages. In the case of FD4, 39201 (oligopeptide transporter 3-like), 37522 (ubiquinol oxidase), 43265 (bidirectional sugar transporter) and 36709 (ABSCISIC ACID-INSENSITIVE 5-like protein) showed higher expressions as compared to other stages of flower development. However, 42571 (LOB domain-containing protein) was fairly high in FD5 than FD4.

In vegetative tissues, 43471 (bidirectional sugar transporter) was exclusively expressed in leaf as compared to other tissues (Figure 8b). In root, 43615 (7-deoxyloganetin glucosyltransferase) and 39560 (MADS-box transcription factor 27) were specifically expressed. However, 38755 (Flavonoid 3′-monooxygenase), and 42857 (Transcription factor MYB39) and 43139 (Protein indeterminate-domain 14) showed higher expressions in capsule instead of root and leaf, respectively. This deviation might be related to the presence of more than one homolog of these genes in our transcriptomic data.

## 3. Discussion

Bamboo orchid is successful in the orchid industry because of its short juvenile phase and continuous flowering pattern. This makes it an ideal plant to study flowering time regulation in orchids where long juvenile phases and short flowering time are major industrial drawbacks, although the floral beauty of orchids is second to none. As a starting point, stage and tissue specific gene mining can provide valuable information on how the flower development is regulated step by step and how other tissues signalize this development. Therefore, current transcriptome study was performed to see the tissue specific gene regulation in five stages of flower development and four tissue types of *A. graminifolia*. Reproducible expression profiles were observed during flower development and the stages or tissues were distinguished from each other in PCA (Figure 1a), suggesting the specific genetic programs for stages of flower development and tissue types. However, FD1 and FD2 were clustered together (Figure 1a) and most of the genes showing high expression in FD1 also showed upregulation in FD2 (Figure 3). Extensive involvement of hormonal regulation and cell activities can be seen in FD1 as compared to late stages of flower development and other tissues (Figure 6), suggesting the involvement of multiple factors in the initiation of flower development.

Gibberellins, cytokinins and auxins are the important promoters of bud growth, while abscisic acid and jasmonic acid are the inhibitors [29,30,31,32,33,34]. A significant number of hormone regulatory genes were found in our transcriptome data. Plant hormone and signal transduction pathway was the most enriched among the KEGG pathways (Figure 2b). The highest number of hormone regulatory DEGs were related to auxin and gibberellin control (Figure 6e), suggesting the significant involvement of growth promoters. Auxins and gibberellins were mainly upregulated in the early stages (FD1 and FD2) of flower development, while ABA was downregulated in the early FD stages. This signifies the expected antagonistic relationships among growth promoters and inhibitors. However, cytokinin-related genes were not as abundant. They were mainly upregulated in the late stages of flower development, leaf and root.

The LAX2 (37931) was highly expressed in FD1 as compared to other stages of flower development. The export and import of auxin from the cell is regulated by auxin efflux and influx carriers, including PIN and LAX, respectively [35]. YAB4 (37740) and GASA3 (36033) are the GA pathways genes and showed high expression in FD1 (Figure 8a). YABBY genes are the family of transcription factors that regulate multiple aspects of vegetative parts and flower development [36]. Overexpression of YABBY4 in rice leads to GA insensitivity and an increased number of floral organs [36]. GA regulates plant growth and development and flowering mainly through DELLA proteins. Therefore, degradation or stabilization of DELLA is key to GA signaling. *OsYABBY4* suppresses *SLR1* and *GA20ox2*. It binds to the promoter region of GA20ox2, which is a target of SLR1, and also physically interacts with SLR1, thereby fulfilling the feedback loop of GA in which GA homeostasis is modulated by *SLR1* through regulating gene expression [36]. GASA genes show tissue and organ specific expressions in *Arabidopsis* (13) and are upregulated by GA [37].

In the late stage of flower development (FD4), SWEET4 (43265) was highly expressed (Figure 8a). AtSWEET4 acts as a hexose facilitator and involves the transport of sugars (glucose and fructose) to different sinks, thereby impacting plant development [38,39]. ABA and actin cytoskeleton are the important regulators of stomatal movement in plants. Actin-depolymerizing factors (ADFs) are involved in actin-bundling activities. DPBF3 is an ABF/AREB transcription factor and involves the transcriptional activation of *ADF5* promoter via ABA-signaling pathway [40]. DPBF3 (36709) was highly expressed in FD4. The *A. thaliana* AtOPT3 is an oligopeptide transporter protein and plays important role in embryo development [41]. Our transcriptome data also identified OPT3 (39201) with the highest expression in FD4 (Figure 8a).

Leaves and roots also showed specificity towards hormonal regulation that may integrate flowering development through multiple pathways. AtSWEET13 is a sugar transporter and involves GA-mediated physiological responses in plants [42]. We found SWEET13 (43471) with the highest expression in leaves (Figure 8b). LONELY GUY (LOG) is a cytokinin-activating enzyme and involves activation pathways in shoot meristems. It showed higher expression in leaf as compared to other tissues (Figure 7). In Arabidopsis, LOGs involve promotion of cell division in leaves and embryos, delaying of leaf senescence and reduction in the apical dominance [43]. Gretchen Hagen3 (*GH3*) is an auxin early response gene that plays an important role in plant development [44]. GH3.1 was highly expressed in leaves (Figure 7). In *Citrus sinensis*, *CsGH3.1* acts as functional IAA-amido synthase to regulate free IAA levels [45]. MADS27 (39560) was only expressed in roots. It is a miRNA target and plays a role in plant development and flowering [46]. The UGT85A24 (43615) was also expressed only in roots. It is a UDP-glucose:iridoid glucotransferase which involves the biosynthesis of iridoids, which are the widely distributed secondary metabolites and possess medicinal value [47].

Therefore, this mining of stage-specific gene profiles shows the variety of gene regulators that play a role in the vegetative and reproductive growth of bamboo orchid. Signaling pathways of major plant hormones are evident from the data cascade, and this digging further validates the importance of *A. graminifolia* in the breeding programs for orchids.

## 4. Materials and Methods

### 4.1. Plant Material and Growth Conditions

The bamboo orchids were grown at the Institute of Environmental Horticulture (GAAS, China) at a temperature of 25/20 °C (day/night) and light duration of 16 h. Samples were collected in three technical and biological repeats from five stages of flower development (FD1–FD5), mature flower, capsule, leaf and root. Collection was made in liquid nitrogen with immediate storage at −80 °C until further use.

### 4.2. Library Preparation and RNA Sequencing

Total RNA was extracted from 9 tissues (5 FD stages and 4 plant parts) and cDNA libraries were prepared. RNA quality and quantity check was made through Nano-Drop spectrophotometer and cDNA libraries were prepared according to Illumina protocol [48]. The mRNA fragments of about 200 bp were obtained from total RNA, and first and second strand cDNAs were prepared and adapter ligation and low-cycle enrichment were performed according to TruSeq^®^RNA HT Sample Prep Kit from Illumina (San Diego, CA, USA). The purified library products were diluted to 10 pM, and sequencing was performed. Finally, de novo transcriptome was done with Trinity program using default parameters [49].

### 4.3. Selection and Analysis of DEGs

Gene expression was ascertained as FPKM values with following formula:

FPKM = [total exon reads/mapped million reads] × exon length (kb)

The edgeR package was used to find significant differences in gene expression and a threshold significance level was set at FDR < 0.05 and log2 ratio of more than one. Principal component analysis (PCA) and Pearson correlation coefficient (PCC) analysis were used to ascertain relationships among different samples. Clustering analysis was performed using corrplot and prcomp packages in R [50].

The differentially expressed genes (DEGs) were annotated to KEGG and GO platforms to learn the involvement of different regulatory pathways or biological process, respectively [51,52]. A *p* or *q*-value of ≤0.05 was set to find significantly enriched KEGG pathways or GO terms.

The stage specific DEGs were blasted into the Arabidopsis genome using Blast-2.10.0+ system and Arabidopsis locus IDs were selected at an Evalue of 1 × e^−5^. The IDs were run in BINGO app using Cytoscape [53] to ascertain the clustering of co-expressed gene sets. Pathway enrichment of stage-specific gene sets was made using MapMan(v3.6.0RC1) (https://mapman.gabipd.org/mapman-version-3.6.0; accessed on 1 September 2021) with TAIR10 (best Arabidopsis homolog) as reference.

### 4.4. Identification of Highly Differential and Stage Specific Gene Sets

The edgeR package of R was used to filter the gene sets with most differential expressions across all the samples. This generated top 500 or 50 highly differential genes among all the DEGs.

Stage-specific/preferentially expressed gene sets were identified using stage specificity (SS) scoring algorithm. This scoring algorithm identifies genes specific to each developmental stage or position by comparing the expressions of genes in a particular stage with their maximum values in other stages [53]. Higher SS score of genes in a specific stage provides the significant expression of those genes at that stage. The stage-specific genes sets were used to make heatmap through ggplot2 utility in R. Moreover, the gene sets were blasted to get *Arabidopsis* locus IDs to perform clustering analysis using “Bingo” application in Cytoscape [53].

### 4.5. Weighted Gene Coexpression Network Analysis (WGCNA)

WGCNA package of R was used, as previously reported [53], to identify coexpressed modules for early (FD1) and late (FD4) stages of flower development, leaf and root. The Cytoscape edges produced by WGCNA were used to select top 5 hubs with maximum connectivity among DEGs.

### 4.6. RT-qPCR Analysis

A total of 16 genes were evaluated through qRT-PCR. Total RNA was extracted from 9 tissues and cDNA was prepared. The 20 µL qRT-PCR mixture contained 2 µL cDNA and 10 µL SYBR premix Ex-taq™ (Takara, Kusatsu, Japan). The qRT-PCR was performed using Bio-Rad CFX-96 RealTime PCR System (Bio-Rad, Hercules, CA, USA). Actin was the internal standard to normalize expressions. The primers used for gene cloning are shown in Supplementary Table S3.

### 4.7. Statistical Analysis

The expression values were statistically tested by one-way ANOVA with SPSS software (SPSS Inc., Chicago, IL, USA; v16.0). The significance is shown at *p* < 0.05 (*) or *p* < 0.01 (**).

## 5. Conclusions

This study explores the genetic protocols related to the growth and development of bamboo orchid (*Arundinagraminifolia*). Stage-specific highly expressed gene sets were found through transcriptomic analysis. This staggering depicted the partition of genomic data specific to hormonal and cell cycle activities for different stages of flower development and tissue types. WGCNA was used to produce clusters of highly co-expressed genes for early and late stages of flower development, leaf and root. Hormonal pathways were mainly involved, and hub hormonal regulators were found for stages of flower development and tissue types. Knowing that *A. graminifolia* completes its vegetative and reproductive cycle much faster than other orchids, the outcomes of this research would be valuable to accelerate the breeding programs for other orchids.

## Data Availability

The relevant data is provided as supplementary materials.

## Supplementary Materials

The following are available online at https://www.mdpi.com/article/10.3390/ijms222010935/s1.
