# Supplementary material for: Stage Specificity, the Dynamic Regulators and the Unique Orchid Arundina graminifolia
Source: Int J Mol Sci. 2021 Oct 10;22(20):10935. doi: 10.3390/ijms222010935 (PMC8535972; doi:10.3390/ijms222010935)

Supplementary Figure S1. Floral organ development and morphogenesis of *A. graminifolia*. (**A**–**C**): Flower bud differentiation period 1; (**D**–**G**): Sepal growth period 2; (**H**–**K**): Pillar development period 3; L-O: Flower dyeing period 4; (**P**–**Q**): Flowering period 5; A: The inflorescence; B: The inflorescence longitudinal section; C: Flower 1 (Fl 1); D, H, L: The whole flower bud; E, I, M: The longitudinal section of the flower bud; F, J, N: The pillar; G, K, O, Q: The organ anatomy; P: The flower; Se: Sepal; LSe: The lateral sepal; DSe: The dosal sepal; LPe: Lateral petal; La: Labe llum; Pi: Pillar; Br: Bract; Fl 1, Fl 2, Fl 3: Flower 1, F lower 2, Flower 3; An: Anther; Ro: Rostellum; St: Stigma; Sty: Style; Anc: Anther cap.

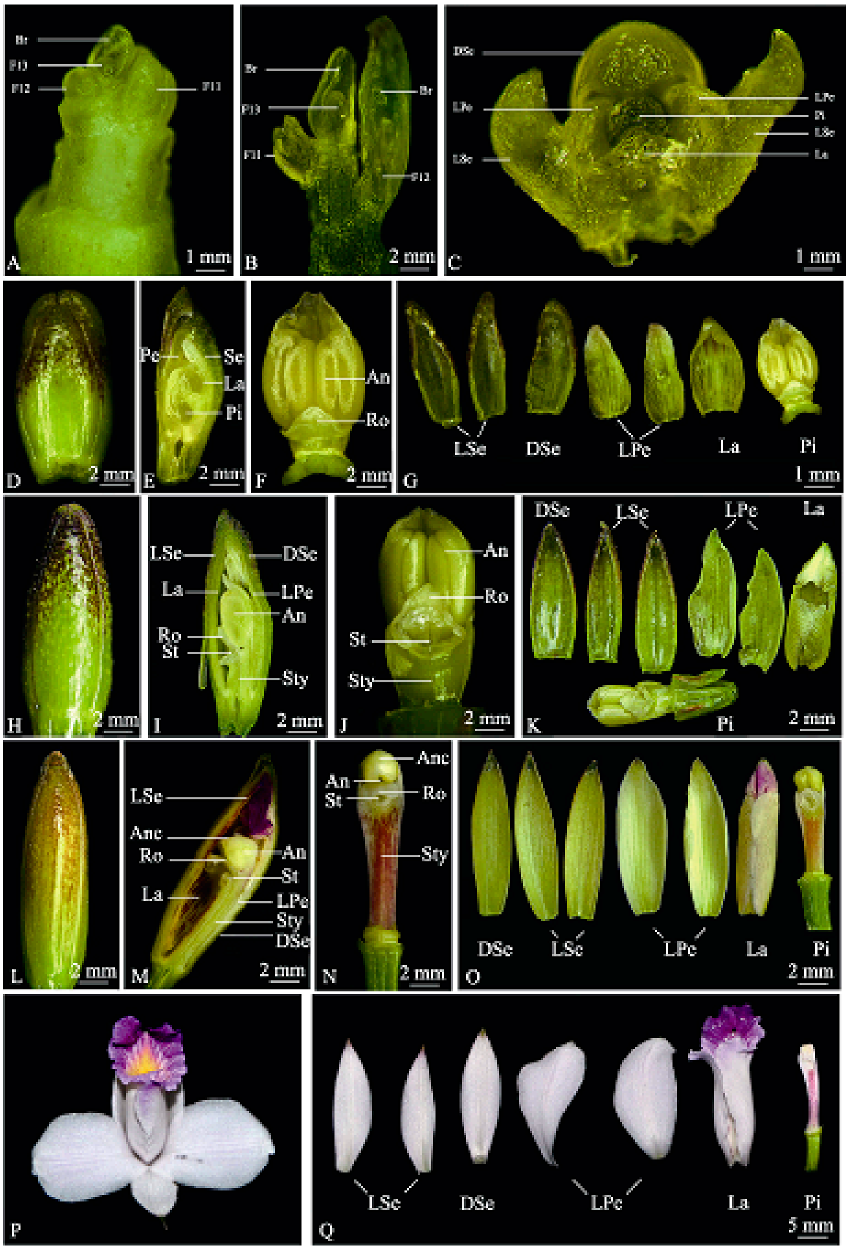

Supplement: Supplementary file 1 [file ijms-22-10935-s001.zip › Supplementary Figures.pdf]
